# Effect of sacrocolpopexy and retropubic sling on overactive bladder symptoms

**DOI:** 10.4274/jtgga.2016.0176

**Published:** 2017-03-01

**Authors:** Muhammad Faisal Aslam, William T. Gregory, Blake Osmundsen

**Affiliations:** 1 Department of Obstetrics and Gynecology, St. John Hospital and Medical Center, Detroit, MI, USA; 2 Department of Obstetrics and Gynecology, Oregon Health&Science University, Portland, OR, USA; 3 Department of Obstetrics and Gynecology, Legacy Good Samaritan Medical Center, Portland, OR, USA

**Keywords:** Overactive bladder, sacrocolpopexy, midurethral sling, transvaginal tape, prolapse repair

## Abstract

**Objective:**

In this study, we aimed to evaluate the effect of sacrocolpopexy and retropubic midurethral sling, or transvaginal tape (TVT) procedure, on overactive bladder (OAB) symptoms. Our null hypothesis was that concomitant sacrocolpopexy and TVT exacerbate OAB symptoms.

**Material and Methods:**

This is a prospective cohort study. All subjects had apical/anterior prolapse and underwent robotic-assisted sacrocolpopexy and TVT, with or without concomitant hysterectomy. All subjects completed a standardized one-year follow-up between 2009 and 2014. To assess for OAB symptoms, we used the Urogenital Distress Inventory subscale questions #15 and/or question #16. Reponses to these questions are based on a five-point 0 to 4 Likert scale (0 represents a negative response or no symptoms, and 4 represents the most problems). Any patient who answered 1 or higher on the Likert scale, either on the frequency or urge incontinence question, was defined as having OAB symptoms.

**Results:**

Sixty-six subjects completed 12 months of visits. Preoperatively, 54 patients (83%) had OAB symptoms, and postoperatively 29 patients (45%) had OAB symptoms (p<0.001). Patients with postoperative OAB had a lower patient global impression of improvement (PGI-I) scores, PGI-I 5.8 with OAB, and PGI-I 6.6 without OAB (p<0.003).

**Conclusion:**

We found that sacrocolpopexy and concomitant retropubic midurethral sling does not contribute to additive OAB symptoms, and symptoms actually resolved in 38% of women in our cohort. The presence of postoperative OAB contributes to lower global impression of improvement.

## INTRODUCTION

Pelvic organ prolapse (POP) and urinary incontinence are common healthcare problems, with one in four adult women in the United States reporting at least one pelvic floor disorder (1). The lifetime risk of pelvic floor surgery is estimated as 11%-19% (2, 3).

Symptoms of POP and overactive bladder (OAB) are often present together. The International Continence Society defines OAB as urgency with or without urge incontinence, usually with frequency and nocturia (4). Hospital- and community-based studies have shown that the prevalence of OAB is higher in women with POP (5, 6). Presence of OAB symptoms have been reported in up to 88% of women with POP (7). The estimated national cost of OAB in the United States was projected to be $76.2 billion in 2015 and $82.6 billion in 2020, which highlights the magnitude of the economic burden (8, 9).

Several studies have shown improvement in symptoms of OAB after POP surgery (10, 11, 12, 13, 14). The majority of these studies included women who underwent repair in the anterior or apical vaginal compartment. Unfortunately, women who underwent a concomitant stress incontinence procedure were excluded out of concern that an incontinence procedure might aggravate OAB symptoms.

OAB subjectively improves in more than 60% of patients undergoing transvaginal tape (TVT) retropubic midurethral sling (15). However, there is little guidance in the literature regarding women who undergo surgery of the apical compartment of the vagina, along with a concomitant incontinence procedure. We aimed to evaluate the effect of apical POP surgery (sacrocolpopexy) and TVT on OAB symptoms.

## MATERIAL AND METHODS

This is a secondary analysis of subjects who participated in a prospective cohort study whose primary aim was to measure the effect that prolapse surgery had on the ability to cure stress urinary incontinence in patients also receiving a TVT (16). The subjects were recruited from two sites with a Female Pelvic Medicine and Reconstructive Surgery fellowship (an academic institution and a community-based institution in the Pacific Northwest of the United States) and followed for one year between January 2009 and January 2014.

All participants had apical/anterior prolapse and underwent robotic-assisted sacrocolpopexy and TVT, with or without concomitant hysterectomy, and completed a standardized one-year follow-up. 

The specific surgical steps were similar at both sites. We used 5 abdominal ports (1 camera arm, 3 robotic arms, and 1 assist port) with the patient in the deep Trendelenburg position. The sacrocolpopexy was started by opening the presacral space to expose the anterior longitudinal ligament at the level of S1. The peritoneal opening was then extended along the right pelvic sidewall into the pouch of Douglas. We performed a deep dissection on both the anterior and posterior vaginal walls to facilitate placement of an adequately sized piece of mesh to address prolapse in all three compartments. We used polyproplylene Gynemesh PS Mesh (Ethicon, USA) for the sacrocolpopexy. We used 4-6 delayed absorbable Maxon sutures (Covidien-Medtronic) anteriorly and 6-8 Maxon sutures posteriorly on the vagina to affix the mesh. The sacral end of the mesh was attached to the anterior longitudinal ligament at the level of S1, with two non-absorbable sutures of 0-Ti-Cron (braided polyester). TVT was performed after the laparoscopic portion of the case had been completed. The TVT procedure was performed as described by Ulmsten et al. (17). Tensioning of the sling was performed by placing Metzenbaum scissors between the sling and the mid urethra until the plastic sheaths were withdrawn.

Inclusion criteria were women with symptomatic prolapse with the leading edge of the prolapse extending beyond the hymen, and symptoms of stress incontinence and documented stress incontinence on urodynamics. All subjects had a complete preoperative evaluation and physical exam, including POP quantification (POP Q) examination, and each completed the validated short form of the Pelvic Floor Distress Inventory (PFDI-20) questionnaire. Informed consent was obtained from all subjects. The subjects subsequently completed 12 months follow-up with a POP Q examination and the same questionnaires, and also completed the Patient Global Impression of Improvement (PGI-I).

Ethics committee approval was obtained from the Institutional Review Board (IRB) at both sites, and we used an IRB approved data repository Filemaker Pro (FileMaker, Inc. CA). Data from both sites were entered into a standardized template in Filemaker. At one year, the subjects returned for a research evaluation. Urogynecology fellows who did not participate in the original surgery conducted the follow-up exams.

To assess for OAB symptoms, we used Urogenital Distress Inventory subscale (UDI) questions #15 (“Do you usually experience frequent urination?”) and/or question #16 (“Do you usually experience urine leakage associated with a feeling of urgency, a strong sensation of needing to go to the bathroom?”). Reponses to these questions are based on a five-point 0 to 4 Likert scale, where 0 represents a negative response or no symptoms, and 4 represents the most problems. Any patient who answered 1 or higher on the Likert scale, either on the frequency or urge incontinence question, was defined as having OAB symptoms.

In order to assess the role of POP Q stages in OAB symptoms following sacrocolpopexy, we also dichotomized the POP Q staged by combining stages 1-2 and 3-4. This was done to perform analysis once prolapse was divided into less severe (stages 1-2) and more severe (stages 3-4) anatomic groups based on the POP Q.

Statistical analysis was performed using SPSS (Version 22.0. Armonk, NY: IBM). Based on prior reports focusing only on POP surgery and its effect on OAB symptoms, we assumed a prevalence of OAB symptoms of about 70% in women with POP, and 50% improvement in symptoms after surgery that included both prolapse repair and surgery for stress incontinence (10, 11, 13). Assuming an alpha of 0.05 and 80% power, we anticipated that 62 subjects would be required. Univariate analysis was performed using Student’s t-test for continuous variables and Chi-square for categorical variables. We used Fisher’s exact test when assumptions for Chi-square distribution were violated. Wilcoxon’s signed-rank test was performed to compare the urinary frequency and urge incontinence scores between the preoperative and postoperative groups, and McNemar’s test was used to compare the OAB symptoms between these groups. We used the Mann-Whitney U test to compare PGI-I scores between those with and without OAB postoperatively.

## RESULTS

We enrolled 77 subjects from both sites. Eleven subjects did not attend the 12 monthly visits and were excluded. Sixty-six subjects completed 12 monthly visits; one had missing data. Sixty-five subjects were included in the final analysis. Our population was predominantly white (96%). Patient characteristics are shown in Table 1.

Prolapse measurements, as examined using the POP Q system, showed significant improvement at one year postoperatively (Table 2).

Preoperative urinary frequency was present in 43 (67%) subjects; 18 (28%) had urinary frequency postoperatively. Preoperatively, 41 (64%) had urge incontinence. In the postoperative group, 25 (39%) subjects reported urge incontinence.

The median preoperative score for urinary frequency was 2.5 and this reduced postoperatively to 0, and was significantly different with a p value of <0.001. The median preoperative score for urge incontinence was 2 and this reduced to 0 postoperatively; this was significantly different with a p value of 0.004.

Preoperatively, 54 (83%) patients had OAB symptoms, and 31 (48%) patients had OAB symptoms postoperatively. This difference was statistically significant with a p value of <0.001 (Table 3). Patients with postoperative OAB had a lower global impression of improvement, PGI-I 5.8 with OAB, PGI-I 6.6 without OAB (p<0.003).

We also looked at de novo OAB symptoms. In our cohort, 54 out of 66 subjects had OAB preoperatively, it resolved in 26 of these subjects postoperatively, showing a 48% reduction in OAB (p<0.001). Of the 12 patients who did not have OAB preoperatively, only five developed it postoperatively, a de novo OAB rate of five out of 66 (7.5%).

In a multivariate logistic regression: age, body mass index (BMI), parity, concomitant hysterectomy, and pre- and postoperative stage of prolapse revealed no significant relationship between these variables and the presence of postoperative OAB (Table 4).

We assessed the role of urodynamics, and 5/66 patients had preoperative detrusor overactivity (DO) on urodynamics. OAB resolved in 3 out 5 (60%) of these patients.

In order to assess the impact of prolapse severity, we dichotomized the POP Q stages by combining stages 1-2 and 3-4. These baseline characteristics are shown in Table 5. Women with stages 1-2 POP were younger than women with stages 3-4 (mean age: 58.8 vs. 69.3 years, p<0.001). There was no difference in BMI and parity.

Thirteen (57%) women in stages 1-2 reported urinary frequency compared with 30 (71%) women in the stage 3-4 group on PFDI-20, which was not statistically different (p=0.25). Similarly, 14 (61%) women in the stage 1-2 group and 27 (64%) women in the stage 3-4 group reported baseline urge incontinence (p=0.86). There was no difference postoperatively between the two groups. Only 4 (17%) women reported urinary frequency from the stage 1-2 group and 14 (32%) in the stage 3-4 group (p=0.18). Postoperatively, urge incontinence was present in 7 (30%) women in the stage 1-2 group and 18 (42%) women in the stage 3-4 group (p=0.35).

## DISCUSSION

The primary aim of our study was to evaluate the effect of sacrocolpopexy and TVT on OAB symptoms. We found that almost half of the patients with OAB symptoms preoperatively had resolution of symptoms at 12 months after surgery. OAB significantly affects the quality of life of women. The National Overactive Bladder Evaluation study found the overall prevalence of OAB in women as 16.9% (18). However, the prevalence of OAB is even higher in women with POP (5, 6), and has been reported to be up to 88% (7). OAB causes a dramatic reduction in quality of life of women (19). Stress, anxiety, and generalized irritability are higher in women with OAB (20). In our cohort, a significant proportion (83%) of women with apical POP and stress incontinence had OAB symptoms. However, postoperatively, only 45% percent of women reported OAB symptoms at one year. We found that sacrocolpopexy and concomitant TVT does not contribute to additive OAB symptoms, and these symptoms resolved in 38% of women.

Prior reports have shown a reduction in OAB symptoms after prolapse surgery (10, 11, 12, 13, 14), though out of concern for aggravation of OAB symptoms, incontinence procedures were excluded from these studies. Although they may have been counseled otherwise, women undergoing surgery for stress incontinence expect their symptoms of OAB to improve as well (21). Recognizing this as a potential source of disappointment, our study found that participants with postoperative OAB had lower global impression of improvement.

Midurethral slings have been shown to improve OAB symptoms in women with mixed incontinence without prolapse (15, 22). Similarly, prolapse repairs alone have shown improvement in OAB symptoms (10, 11, 12, 13, 14). However, whether that same effect can be seen in women receiving slings and undergoing complex prolapse surgery is not as well defined. One of the reasons for this concern is that by adding the incontinence sling procedure at the time of prolapse surgery, we are adding another variable that might exacerbate OAB symptoms. However, we found that OAB symptoms improved, despite the concomitant TVT procedure. We also studied urinary frequency and urgency/urge incontinence separately and found improvement in both facets. Almost half of the patients with OAB symptoms preoperatively had resolution of symptoms at 12 months after surgery. The incidence of de novo OAB was only 7.5% in our cohort, with only 5 patients developing de novo OAB at 12 months.

In our cohort, 5/66 patients had preoperative DO on urodynamics, and symptoms resolved in 3 out 5 (60%) of these patients. Although our numbers are small, we found that presence of DO on urodynamics might not confer persistent OAB symptoms postoperatively.

We also dichotomized the preoperative prolapse stage as stages 1-2 and 3-4. We found no difference in baseline urinary frequency and urge incontinence in the two groups. Furthermore, we found no difference in OAB symptoms postoperatively based on preoperative stage.

Most prior studies included women undergoing mixed prolapse surgeries or anterior vaginal wall repairs. Our cohort included women who underwent sacrocolpopexy and TVT (with or without concomitant procedures). It is possible that the symptoms improved because POP causes obstruction of the urethra, which results in OAB symptoms in women, similar to benign prostatic hypertrophy causing OAB symptoms in men (23). Petros and Ulmsten (24) proposed the “integral theory,” in which they suggested that the anterior vaginal wall relaxation was associated with OAB symptoms. It is plausible that women with prolapse, stress incontinence, and OAB do not have two separate conditions, and the symptoms of mixed urinary incontinence are a spectrum of stress urinary incontinence. This could explain the improvement of OAB symptoms in women undergoing concomitant incontinence sling and prolapse repair.

To our knowledge, this is the first study to assess the effect of concomitant sacrocolpopexy and TVT on OAB symptoms. The strengths of our study are that it is a prospective study, and we used validated questionnaires to assess for OAB symptoms. Everyone in our cohort underwent sacrocolpopexy, which is widely considered as the gold standard procedure for apical prolapse. A limitation of our study is the lack of urodynamic data for the postoperative assessment of resolution of DO. However, we used validated questionnaires and urodynamics have a low predictive value to reproduce clinical findings of OAB (25). Another limitation is that we did not account for concurrent medication use in these subjects. In conclusion, in this prospectively recruited cohort of women, sacrocolpopexy and concomitant TVT did not contribute to additive OAB symptoms, which actually resolved in almost half of the women. The presence of postoperative OAB contributes to lower global impression of improvement. These findings will be helpful to providers, and will assist in counseling patients.

## Figures and Tables

**Table 1:**
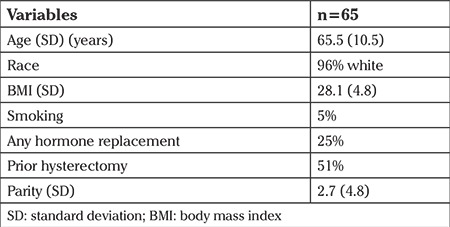
Baseline demographics

**Table 2:**
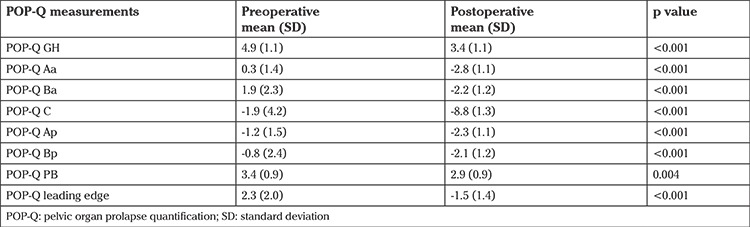
Prolapse measurements

**Table 3:**
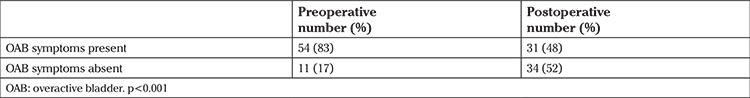
Preoperative and postoperative overactive bladder symptoms

**Table 4:**
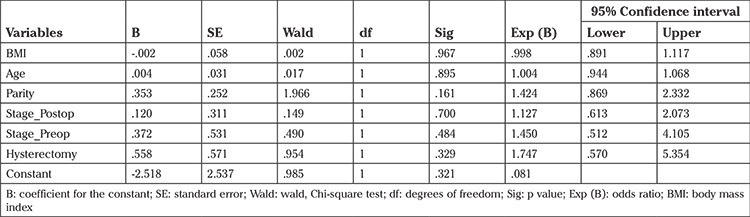
Multivariate logistic regression

**Table 5:**
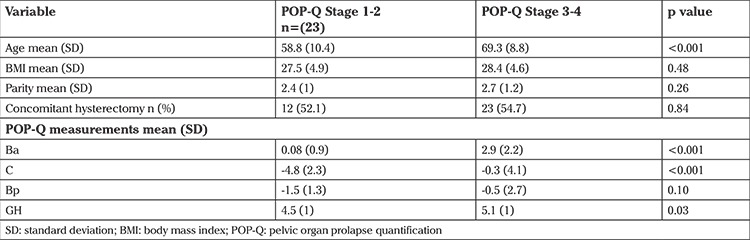
Baseline characteristics after dichotomizing stages

## References

[1] Wu JM, Vaughan CP, Goode PS, Redden DT, Burgio KL, Richter HE, et al (2014). Prevalence and trends of symptomatic pelvic floor disorders in U. S. women. Obstet Gynecol.

[2] Smith FJ, Holman CD, Moorin RE, Tsokos N (2010). Lifetime risk of undergoing surgery for pelvic organ prolapse. Obstet Gynecol.

[3] Olsen AL, Smith VJ, Bergstrom JO, Colling JC, Clark AL (1997). Epidemiology of surgically managed pelvic organ prolapse and urinary incontinence. Obstet Gynecol.

[4] Abrams P, Cardozo L, Fall M, Griffiths D, Rosier P, Ulmsten U, et al (2002). The standardisation of terminology of lower urinary tract function: report from the Standardisation Sub-committee of the International Continence Society. Am J Obstet Gynecol.

[5] Boer TA, Salvatore S, Cardozo L, Chapple C, Kelleher C, Kerrebroeck P, et al (2010). Pelvic organ prolapse and overactive bladder. Neurourol Urodyn.

[6] Lawrence JM, Lukacz ES, Nager CW, Hsu JW, Luber KM (2008). Prevalence and co-occurrence of pelvic floor disorders in community-dwelling women. Obstet Gynecol.

[7] Digesu GA, Chaliha C, Salvatore S, Hutchings A, Khullar V (2005). The relationship of vaginal prolapse severity to symptoms and quality of life. BJOG.

[8] Coyne KS, Wein A, Nicholson S, Kvasz M, Chen CI, Milsom I (2014). Economic burden of urgency urinary incontinence in the United States: a systematic review. J Manag Care Pharm.

[9] Ganz ML, Smalarz AM, Krupski TL, Anger JT, Hu JC, Wittrup-Jensen KU, et al (2010). Economic costs of overactive bladder in the United States. Urology.

[10] Basu M, Wise B, Duckett J (2013). Urgency resolution following prolapse surgery: is voiding important?. Int Urogynecol J.

[11] Miranne JM, Lopes V, Carberry CL, Sung VW (2013). The effect of pelvic organ prolapse severity on improvement in overactive bladder symptoms after pelvic reconstructive surgery. Int Urogynecol J.

[12] Lensen EJ, Withagen MI, Kluivers KB, Milani AL, Vierhout ME (2013). Urinary incontinence after surgery for pelvic organ prolapse. Neurourol Urodyn.

[13] Boer TA, Kluivers KB, Withagen MI, Milani AL, Vierhout ME (2010). Predictive factors for overactive bladder symptoms after pelvic organ prolapse surgery. Int Urogynecol J.

[14] Digesu GA, Salvatore S, Chaliha C, Athanasiou S, Milani R, Khullar V (2007). Do overactive bladder symptoms improve after repair of anterior vaginal wall prolapse?. Int Urogynecol J Pelvic Floor Dysfunct.

[15] Duckett JR, Tamilselvi A (2006). Effect of tension-free vaginal tape in women with a urodynamic diagnosis of idiopathic detrusor overactivity and stress incontinence. BJOG.

[16] Osmundsen B, Gregory WT, Denman MA, Adams K, Edwards R, Clark A (2015). Tension-Free Vaginal Tape Failure After Robotic Sacrocolpopexy and Tension-Free Vaginal Tape for Concomitant Prolapse and Stress Incontinence. Female Pelvic Med Reconstr Surg.

[17] Ulmsten U, Henriksson L, Johnson P, Varhos G (1996). An ambulatory surgical procedure under local anesthesia for treatment of female urinary incontinence. Int Urogynecol J Pelvic Floor Dysfunct.

[18] Stewart WF, Van Rooyen JB, Cundiff GW, Abrams P, Herzog AR, Corey R, et al (2003). Prevalence and burden of overactive bladder in the United States. World J Urol.

[19] Dubeau CE, Simon SE, Morris JN (2006). The effect of urinary incontinence on quality of life in older nursing home residents. J Am Geriatr Soc.

[20] Chiara G, Piccioni V, Perino M, Ohlmeier U, Fassino S, Leombruni P (1998). Psychological investigation in female patients suffering from urinary incontinence. Int Urogynecol J Pelvic Floor Dysfunct.

[21] Wagg A, Das Gupta R, Assassa P, Shaw C, Mayne C, Martin M (2005). Secondary-care treatment patterns in the UK for women with urinary incontinence. BJU Int.

[22] Han JY, Choo MS, Lee YS, Seo JT, Kim JH, Kim YH, et al (2013). Effectiveness of retropubic tension-free vaginal tape and transobturator inside-out tape procedures in women with overactive bladder and stress urinary incontinence. Int Neurourol J.

[23] Kwon JK, Han JH, Choi HC, Kang DH, Lee JY, Kim JH, et al (2016). Clinical significance of peripheral zone thickness in men with lower urinary tract symptoms/benign prostatic hyperplasia. BJU Int.

[24] Petros PE, Ulmsten UI (1990). An integral theory of female urinary incontinence. Experimental and clinical considerations. Acta Obstet Gynecol Scand Suppl.

[25] Caruso DJ, Kanagarajah P, Cohen BL, Ayyathurai R, Gomez C, Gousse AE (2010). What is the predictive value of urodynamics to reproduce clinical findings of urinary frequency, urge urinary incontinence, and/or stress urinary incontinence?. Int Urogynecol.

